# Reporting of Artificial Intelligence Diagnostic Accuracy Studies in Pathology Abstracts: Compliance with STARD for Abstracts Guidelines

**DOI:** 10.1016/j.jpi.2022.100091

**Published:** 2022-02-18

**Authors:** Clare McGenity, Patrick Bossuyt, Darren Treanor

**Affiliations:** a Leeds Teaching Hospitals NHS Trust, Leeds, UK; b University of Leeds, Leeds, UK; c Department of Epidemiology & Data Science, Amsterdam University Medical Centres, University of Amsterdam, Amsterdam, The Netherlands; d Department of Clinical Pathology and Department of Clinical and Experimental Medicine, Linköping University, Linköping, Sweden; e Centre for Medical Image Science and Visualization (CMIV), Linköping University, Linköping, Sweden

## Abstract

Artificial intelligence (AI) research is transforming the range tools and technologies available to pathologists, leading to potentially faster, personalized and more accurate diagnoses for patients. However, to see the use of tools for patient benefit and achieve this safely, the implementation of any algorithm must be underpinned by high quality evidence from research that is understandable, replicable, usable and inclusive of details needed for critical appraisal of potential bias. Evidence suggests that reporting guidelines can improve the completeness of reporting of research, especially with good awareness of guidelines. The quality of evidence provided by abstracts alone is profoundly important, as they influence the decision of a researcher to read a paper, attend a conference presentation or include a study in a systematic review.

AI abstracts at two international pathology conferences were assessed to establish completeness of reporting against the STARD for Abstracts criteria. This reporting guideline is for abstracts of diagnostic accuracy studies and includes a checklist of 11 essential items required to accomplish satisfactory reporting of such an investigation. A total of 3488 abstracts were screened from the United States & Canadian Academy of Pathology annual meeting 2019 and the 31st European Congress of Pathology (ESP Congress). Of these, 51 AI diagnostic accuracy abstracts were identified and assessed against the STARD for Abstracts criteria for completeness of reporting. Completeness of reporting was suboptimal for the 11 essential criteria, a mean of 5.8 (SD 1.5) items were detailed per abstract. Inclusion was variable across the different checklist items, with all abstracts including study objectives and no abstracts including a registration number or registry. Greater use and awareness of the STARD for Abstracts criteria could improve completeness of reporting and further consideration is needed for areas where AI studies are vulnerable to bias.

## Background

Evidence-based medicine is the foundation of good clinical practice, the goal of all health researchers and should underpin the care of every patient. However, despite this shared understanding and ambition, many challenges remain in replicating published evidence across a range of medical fields.^1^, ^2^, ^3^, ^4^, ^5^, ^6^ Specifically, “hot” scientific areas where numerous teams are competing to publish research quickly are particularly at risk of this.^1^^,^^7^^,^^8^ Artificial intelligence (AI) for health applications has generated much excitement, as well as news headlines in recent years.^9^^,^^10^ AI in pathology is a growing area, with increasing numbers of studies appearing in journals and conferences each year.^11^^,^^12^

The Equator Network is an international umbrella organization of professionals working in health research, aiming to improve the reliability, transparency and accuracy of reporting in health literature.^13^ The Network collects reporting guidelines with the goal of making published research: readable, replicable, usable by clinicians in decision making and easy to include in a systematic review.^14^ Reporting guidelines can be found at the website https://www.equator-network.org^15^ and examples include the CONSORT guidelines for randomized trials and the STARD guidelines for diagnostic accuracy studies.^16^^,^^17^ There is evidence that such guidelines have a positive impact on the completeness of reporting of research,^18^^,^^19^ but awareness by researchers and adoption by journals are crucial in determining their success.

Clinicians and researchers may sometimes need to review and make decisions about studies quickly or in large volumes, by simply viewing abstracts. Abstracts play an important role in systematic reviews, where the selection of potentially eligible studies is typically based on reading titles and abstracts. Abstracts also help the reader to decide to read a full article or to attend a conference presentation for example. In recognition of the significance of abstracts in health research, a targeted extension to the STARD reporting guidelines was released in 2017 for conference and journal abstracts.^20^ Eleven essential components were outlined for inclusion in an abstract. The authors demonstrated that this is achievable with the typical 200–300 word count limit.

Finally, whilst current guidelines cover some aspects of the information required to critically appraise AI studies, there remain areas of potential bias that are not adequately addressed.^21^^,^^22^ This has prompted the development of a wave of new extensions and guidelines specific to AI that aim to tackle this problem, such as CONSORT-AI, SPIRIT-AI, STARD-AI, TRIPOD-AI and DECIDE-AI.^22^, ^23^, ^24^, ^25^, ^26^

The relative novelty for many authors, readers, editors and conference attendees of AI studies, combined with the landscape of rising numbers presented at conferences and published in journals, were our rationale for undertaking this evaluation at this time. In this study, the completeness of reporting against the STARD for Abstracts checklist was assessed for AI diagnostic accuracy studies at two international pathology conferences. The aim was to assess current reporting standards of pathology AI diagnostic tools against this guideline. To the best of the authors’ knowledge, whilst both conferences provide guidance on the general structure of the abstract, neither have adopted the STARD for Abstracts checklist or other reporting guidelines as part of their abstract submission process. This study also aims to highlight to researchers the availability of these guidelines for use as simple templates to improve quality and completeness of reporting of research. Finally, a further aim was to identify potential areas of bias in need of additional consideration in the context of AI studies.

## Methods

**Figure 1:**
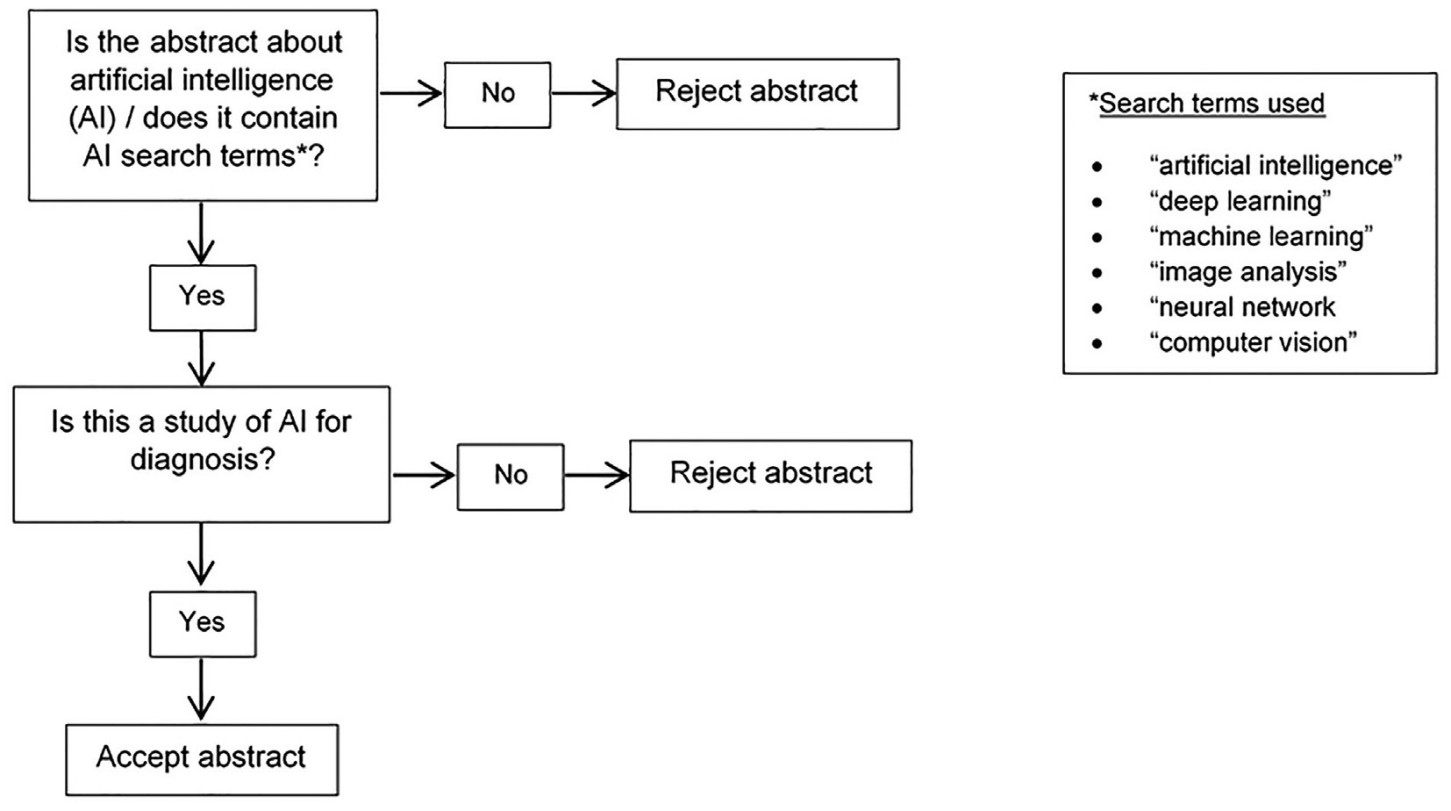
Flowchart of process for identification of artificial intelligence abstracts.

Abstracts were identified from two international pathology conferences. Abstracts from the United States & Canadian Academy of Pathology (USCAP) annual meeting 2019 were available from Modern Pathology via https://www.nature.com/modpathol/articles?type=abstracts-collection&year=2019.^27^ Abstracts for the 31st European Congress of Pathology were available through Virchow’s Archiv via https://link.springer.com/article/10.1007/s00428-019-02631-8.^28^ One reviewer (CM) identified abstracts using the process shown in Fig. 1. The documents were available as PDF files and were searched for key terms using the electronic search function. Additionally, manuscript titles were screened manually for potentially relevant, missed abstracts.

**Figure 2:**
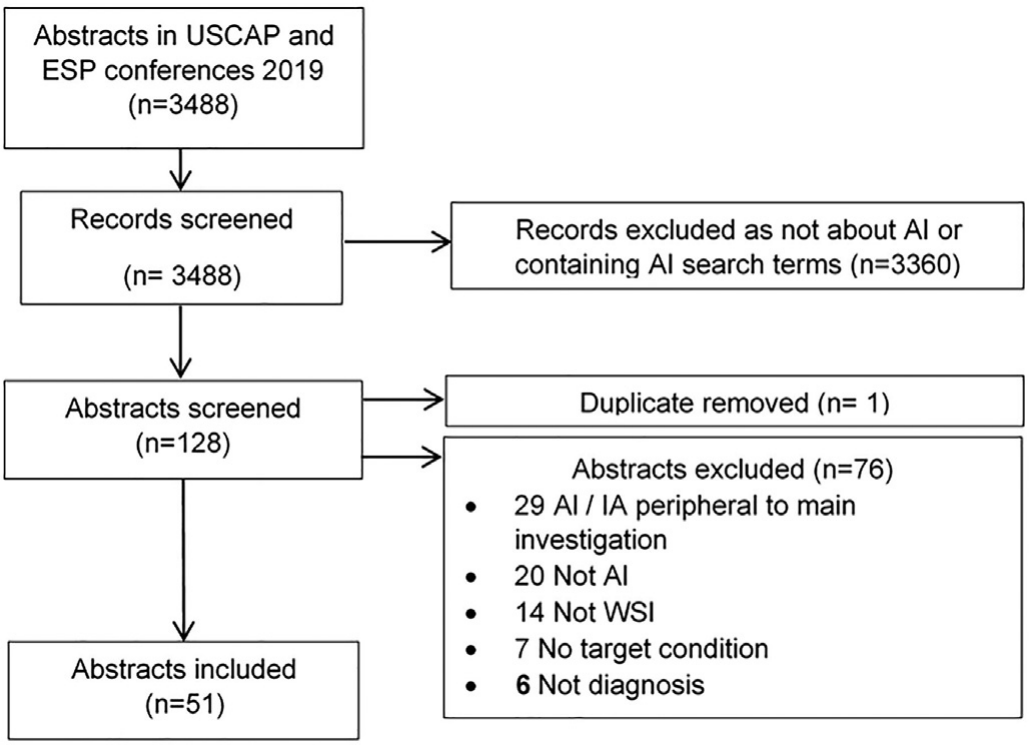
Flowchart of screening process to identify diagnostic accuracy studies of artificial intelligence.

Abstracts describing studies of diagnostic accuracy were identified and Fig. 2 outlines this process.

The inclusion criteria for abstracts were:

• Studies of AI for diagnosis of a target condition.
• Studies using whole-slide imaging (WSI).

The exclusion criteria for abstracts were:

• AI use is peripheral to main investigation.
• The aim of the investigation performed is too ambiguous to be assessed.

Where there was uncertainty in whether to include an abstract, a second checker (DT) was consulted and a consensus decision reached.

Each of the 51 abstracts was assessed against the 11 STARD for Abstracts items as shown in Table 1.^20^ Scoring of each abstract was performed by one reviewer (CM) with a second checker (DT) used for any cases of uncertainty. Additionally, before starting the scoring process, both reviewers (CM and DT) practiced scoring a sample of five abstracts independently and compared results to ensure agreement in the approach to this task.

**Table 1:** The STARD for Abstracts criteria as outlined by Cohen et al. 2017.^20^

The STARD for abstracts 11 checklist items
1. Identification as a study of diagnostic accuracy using at least one measure of accuracy (such as sensitivity, specificity, predictive values or AUC)
2. Study objectives
3. Data collection: whether this was a prospective or retrospective study
4. Eligibility criteria for participants and the settings where the data were collected
5. Whether participants formed a consecutive, random, or convenience series
6. Description of the index test and reference standard
7. Number of participants with and without the target condition included in the analysis
8. Estimates of diagnostic accuracy and their precision (such as 95% confidence intervals)
9. General interpretation of the results
10. Implications for practice, including the intended use of the index test
11. Registration number and name of registry

### Ethical Approval

This study examines previously published data and does not include any new human data or tissue that require ethical approval and consent. The authors assume that the studies examined were conducted after ethical approval and consent, and in accordance with the Declaration of Helsinki.

## Results

Of 3488 abstracts from the USCAP annual meeting 2019 and 31st European Congress of Pathology, 128 contained AI key terms and, of these, 51 were identified as AI studies of diagnostic accuracy. Abstract topics included diseases from nine pathological subspecialty areas with gastrointestinal pathology, breast pathology and urological pathology being the most common (Table 2*)*. There were a range of tasks performed by AI algorithms in the studies such as classification (e.g., between subtypes of breast cancer), detection (e.g., identifying mitotic figures in the tissue), prediction (e.g., likely response to a treatment), segmentation (e.g., outlining tumor against background tissue) and explainability (e.g., applying heatmaps to show where an algorithm has reached decisions). It is worth noting that some abstracts included additional, secondary AI tasks. Classification was the most commonly reported AI task with 20 of 51 abstracts demonstrating this, followed by detection and prediction tasks with 11 of 51 and 10 of 51 abstracts, respectively. In one abstract, the investigation was not completed at the time of abstract submission to the conference and so the nature of the task performed was not clearly described.

**Table 2:** Distribution of abstracts included in final assessment by pathological subspecialty of study.

Pathological subspecialty of study	No. of abstracts	Percent (%)
Gastrointestinal Pathology	13	26
Breast Pathology	11	21
Urological Pathology	9	18
Cardiothoracic pathology	7	14
Dermatopathology	3	5.9
Gynaecological pathology	3	5.9
Haematopathology	3	5.9
Nephropathology	1	2.0
Neuropathology	1	2.0

Heterogeneity was observed in the way dataset numbers were presented across the abstracts. Dataset information was provided at the slide or partial slide level (e.g., glass slides pre-scanning, WSI, patches), at the patient level (e.g., cases or patients) and at the specimen level (e.g., biopsies, tumors). In some instances, a single dataset type was provided, whereas others contained a combination of these or indeed no description of the total dataset numbers at all. It was most common to include dataset totals with patient level data (24 of 51 abstracts), such as number of cases or number of patients with a condition. Table 3 provides a summary of the dataset free-text descriptions included from the abstracts, demonstrating the variation in the presentation of these details across different investigations.

**Table 3:** Summary of dataset descriptions provided by each abstract.

Abstract number	Summarized dataset information provided in each abstract	Abstract number	Summarized dataset information provided in each abstract
1	1522 H&E images, 64 cases	27	410 patients, 1136 biopsy instances
2	805 H&E images	28	443 cropped images from 580 WSIs, 129 biopsies, 129 patients
3	>1288 WSIs (exact total no. not specified)	29	35 slides for training, 80 cases for testing
4	28 patients	30	1461 biopsies, 238 patients
5	1500 cases	31	252 cases, 385 slides
6	115 H&E images	32	1000 cases
7	63 WSIs	33	53 cases
8	532 glass slides, 2162 biopsies	34	266 patients
9	54,587 pixel patches	35	100 cases
10	58 cases	36	225 cases
11	Dataset numbers not given	37	173 WSI
12	19 H&E images	38	417 biopsies
13	23 patients, 23 WSIs, 23 specimens	39	250 cases
14	Dataset numbers not given	40	55,000 patches, 50 cases
15	50 WSIs	41	13 patients
16	3858 patients + 867 external samples	42	60 slides
17	Dataset numbers not given	43	90 tumours
18	58 cases for training, 29 WSIs for testing (exact total no. not specified)	44	232 patients
19	36 biopsies	45	Dataset numbers not given
20	765 biopsy sections	46	Dataset numbers not given
21	300 cases	47	73 WSI
22	1294 WSIs	48	19 slides
23	108 patients	49	156 WSI
24	24 cases, 221 H&E images	50	184 images
25	Dataset numbers not given	51	182,590 patches, 170 patients, 400 biopsies
26	21 cases		

**Figure 3:**
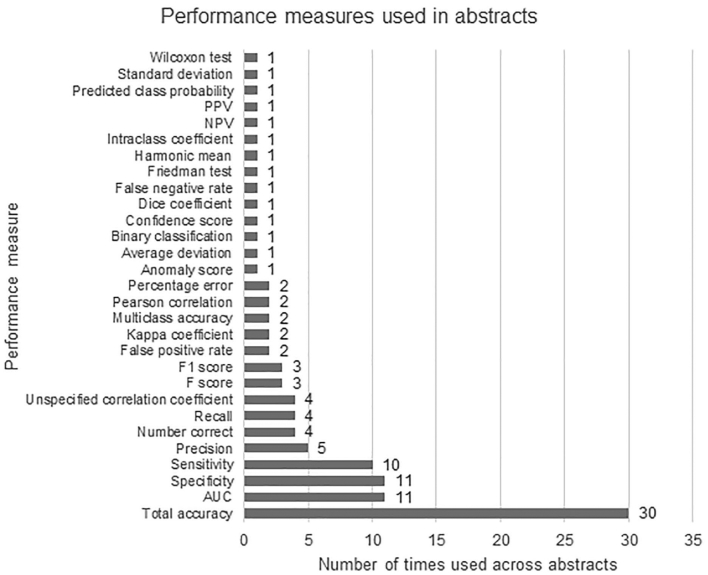
Graph showing the type and frequency of statistical performance measure used in abstracts selected for final assessment.

A range of performance measures were used across the abstracts to express the accuracy of the AI model(s) and these are summarized in Fig. 3. A total of 30 different statistical measures were identified between the 51 abstracts. The most commonly used measures were total accuracy (30 of 51 abstracts), followed by area under the curve (AUC) and specificity (both 11 of 51 abstracts) and sensitivity (10 of 51 abstracts). The number of measures detailed per abstract ranged from 1 to 6, with a mean of 2.1 and median of 2.0 used per abstract.

The 11 STARD for Abstracts checklist items comprise a description and example text to guide the user. These include areas such as the objectives of the study, data collection method, index test and reference standard assessed, interpretation or results and registration of the study. Reporting of these individual checklist items across this study is shown in Table 4. Abstracts performed well in the categories of “identification as a study of diagnostic accuracy” and “study objectives”, with all abstracts including the two items. However, only 8 of 51 of studies included estimates of diagnostic accuracy with their extent of statistical imprecision. Eligibility criteria and setting for data collection, as well as describing the series type for collection were similarly poorly reported with 9 of 51 and 8 of 51 abstracts including these details, respectively. Fewer than half of the studies described whether their investigation was prospective or retrospective (20 of 51 abstracts). A number of abstracts did not provide descriptions of the number of participants with and without the target condition and details of the index test and reference standard, with 28 of 51 and 35 of 51 abstracts giving these details, respectively. Registration number and name of registry was not provided in any of the abstracts.

**Table 4:** Completeness of reporting of abstracts against STARD for Abstracts criteria^20^ by numbers and percentages of abstracts.

STARD for Abstracts checklist item	No. (%) abstracts
1. Identification as a study of diagnostic accuracy using at least one measure of accuracy (such as sensitivity, specificity, predictive values or AUC)	51 (100)
2. Study objectives	51 (100)
3. Data collection: whether this was a prospective or retrospective study	20 (39)
4. Eligibility criteria for participants and the settings where the data were collected	9 (18)
5. Whether participants formed a consecutive, random, or convenience series	8 (16)
6. Description of the index test and reference standard	35 (69)
7. Number of participants with and without the target condition included in the analysis	28 (55)
8. Estimates of diagnostic accuracy and their precision (such as 95% confidence intervals)	8 (16)
9. General interpretation of the results	42 (82)
10. Implications for practice, including the intended use of the index test	42 (84)
11. Registration number and name of registry	0 (0)

**Figure 4:**
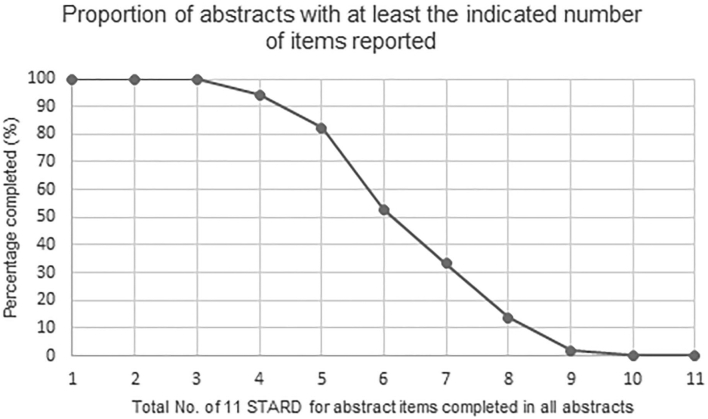
Graph showing the proportion of abstracts with between 1 and 11 of the STARD for Abstracts checklist items provided.

Figure 4 shows the number of studies that provided any of the items from the STARD for Abstracts criteria, ranging from 1 to 11 total checklist items. All abstracts included at least three items from the checklist. No abstracts included 10 or 11 items, and only 2% (1 of 51) abstracts completed 9 items. Furthermore, the mean number of checklist items completed per abstract was 5.8, with a standard deviation of 1.5 and median of 6. The range of items completed per abstract was 3–9. Compliance with the STARD for Abstracts criteria at both the USCAP annual meeting 2019 and the 31st European Congress of Pathology conferences was suboptimal, with key pieces of information often missing across the study reports. This potentially makes it more challenging for clinicians or researchers to rigorously appraise such investigations and to make further use of their findings.

## Related Work

Examples can be seen in the literature for multiple medical fields, where the quality of reporting of diagnostic accuracy studies has been reviewed. Radiology has seen success in the development of image-based AI and consequently, the specialty is likely to see implementation of such tools into clinical practice.^29^ As such, the most pertinent to our present study was an investigation by Dratsch et al. examining radiology AI studies at the European Congress of Radiology for compliance with the STARD for Abstracts guidelines (ECR).^30^ They concluded that overall adherence with the STARD for Abstracts criteria was poor and called for provision of this checklist for all authors submitting abstracts to radiology conferences of diagnostic accuracy studies. STARD for Abstracts had not been adopted before their study but the authors reported that it was planned for use in the ECR 2020 conference. Within the field of pathology, Hogan et al. examined manuscripts from pathology journals for compliance with STARD 2015 guidelines.^31^ They found incomplete reporting of the criteria and suggested that better enforcement of these guidelines was needed to improve standards. Furthermore, before the introduction of the STARD for Abstracts extension in 2017, Korevaar et al. examined the reporting of all diagnostic accuracy studies at an ophthalmology conference and highlighted the crucial information frequently missing in abstracts, creating difficulty when trying to assess risk for bias and applicability to a clinical setting.^32^

However, there is evidence that the STARD guidelines can improve the reporting of diagnostic accuracy studies. In 2011, Selman et al. demonstrated an improvement of adherence to STARD criteria in reporting of diagnostic tests for conditions in Obstetrics and Gynecology following the introduction of the original guidelines released in 2003.^33^ In 2014, Korevaar et al. performed a systematic review and meta-analysis of quality of diagnostic accuracy studies and found a small improvement following the introduction of STARD.^34^ The following year, Korevaar et al. examined relevant studies from 12 high impact-factor medical journals and found there had been a gradual improvement in reporting since the guideline’s introduction.^35^ Most recently, Hong et al. in 2017 assessed radiology diagnostic accuracy studies and found that higher impact-factor journals and “STARD adopter” journals were associated with greater levels of adherence to the STARD criteria.^36^ This would suggest that the STARD guidelines are encouraging better reporting of these investigations where there is awareness and adoption by stakeholders.

## This Study

To assess the internal validity of an AI study, understanding key information about the way the investigation was undertaken is essential. For example, inadequate descriptions of methods of data collection and participants means that confounding factors could be present in the study without the knowledge of the reader, potentially leading to inaccurate conclusions about the AI tool. Furthermore, failing to include details of participants and setting where data was collected could mean that selection bias is present but cannot be assessed.

Similarly to our findings, Korevaar et al. found the reporting of how and where participants were selected and sampling methods in ophthalmology abstracts was poor.^32^ They further identified that less than half of studies reported the reference standard which is in keeping with our findings. Moreover, Dratsch et al. identified poor reporting of data collection, eligibility criteria, type of series and number of participants in radiology AI abstracts, areas of which were poorly reported in our evaluation.^30^

It is difficult to judge the applicability of a test to wider populations if details of the original population sample and how they were selected are unknown to the reader. In our assessment, information was missing for the number of participants with and without the target condition in just under half of the studies, few depicted the eligibility criteria for inclusion of participants and less than half of all abstracts declared whether the study was prospective or retrospective (Table 4*)*.

The number of participants with or without a target condition was well described in many cases but some investigations detailed the number of slides or the number of biopsies instead, and so information at patient level could not be appreciated. Reporting of eligibility criteria and study setting was deficient in many cases as not enough detail was given. Some abstracts included a minimal amount of information but this was not sufficient for the reader to gain a clear picture of the participants and setting(s) of data collection. Minimum requirements for this checklist item are characterized in further detail in the STARD for Abstracts guideline and include whether adults or children were included, proportions of males and females, ages of participants and whether the study was carried out across a single or multiple centers.^20^

It is unclear why the type of data collection was not declared in many abstracts. This is an important point to understand, as retrospective studies of AI are recognized to be at higher risk of bias and prospective evaluation of a test is needed before clinical implementation can realistically be considered.^37^, ^38^, ^39^ We surmise that a prompt to authors to include these details could conceivably improve reporting.

Descriptions of both the index test and reference standard were missing from 16 abstracts (Table 4*)*. In the context of pathology AI, an index test could be an algorithm performing diagnosis and the reference standard could be annotations representing diagnostic features on a digital slide as labeled by a pathologist. Without the incorporation of these components, it becomes impossible to assess if the test was appropriate for the hypothesis. Moreover, reporting of estimates of diagnostic accuracy and their precision were given less frequently, with only eight abstracts adequately detailing this item (Table 4*)*. Whilst many investigations presented a measure of performance, these were rarely accompanied by an indication of their precision (e.g., 95% confidence intervals) which therefore limits the interpretation of the statistical uncertainty and clinical significance of the results. Lastly, the registration number and name of registry was not included in any abstract (Table 4). There are multiple reasons to encourage prospective registration of diagnostic accuracy studies, including avoiding the selective reporting of outcomes, preventing duplication of research, encouraging collaboration and easier identification of unpublished work. In their 2017 paper “Facilitating prospective registration of diagnostic accuracy studies: A STARD initiative”, Korevaar et al. summarized the rationale for registration, as well as practical information on how and where to register a study.^40^

This study highlights variation in the reporting of two areas in particular: the descriptions of datasets and the use of suitable performance measures. Input data were presented in a range of ways with studies giving slide/part of slide-level information, patient-level information, specimen-level information or a combination of these (Table 3). It is challenging to assess the development and evaluation of an AI algorithm when it is unknown if slides are from few or many patients, or if cases provided single or multiple slides for instance. To tackle this problem, authors could be encouraged to provide brief information at both the patient and image level when reporting this type of study. Furthermore, the choice of statistical performance measure varied between abstracts with many different types identified (Fig. 3). This may be in part due to the statistical measures that can be readily obtained from the AI software available to the researcher. However, this heterogeneity presents problems when trying to compile or compare studies, e.g. when conducting a systematic review. Many studies included the use of total accuracy as their choice of measure, making it easier to compare and contrast with other investigations. With the growing number of AI tools in development, areas such as these could be considered when formulating future updates or extensions to reporting guidelines.

We have discussed that prior evidence demonstrates that increasing the awareness and adoption of reporting guidelines can help with the quality of information presented in abstracts and manuscripts for studies of diagnostic accuracy. Adherence to these minimum standards, especially at an early point in an investigation, could conceivably increase the quality of study design in turn. Our investigation shows that reporting in this field is currently lacking and therefore more work is needed to generate widespread knowledge of this guidance to improve standards. Finally, if this guidance was endorsed routinely at pathology conferences, then our analysis could act as a baseline at which to compare a change in reporting quality in the future.

## Discussion

A key intention of this work is to highlight the existence of reporting guidelines and their benefits to the researcher and wider research community. Reporting guidelines can be used as a helpful template to ensure inclusion of all the essential information needed for reporting each study type.^14^, ^15^, ^16^, ^17^ In the example in this study, the effort to write an abstract using the STARD for Abstracts guidelines has been proven to be very achievable within the usual 200–300 word count, and can be performed using the guide shown within the original STARD for Abstracts paper.^20^ Evidence shows that reporting guidelines can improve completeness of reporting and that the endorsement of their use does not hinder completeness of reporting.^19^^,^^33^, ^34^, ^35^ Complete reporting can help other researchers to understand the design of the study, to appraise the methodological rigor and to accurately interpret and appraise the findings of the research.^16^^,^^17^ Thorough and transparent reporting can assist replication of research, impact other work in the same field and ultimately benefit patients with high quality research outputs.^16^^,^^17^^,^^19^ The STARD reporting guidelines were developed with the assistance of multiple major clinical journals, which also require their use in the reporting of diagnostic accuracy studies.^17^^,^^20^ There may be the perception that reporting guidelines create additional work for researchers. However, as shown, there are benefits to researchers who use them, and they can reduce research waste and increase quality and replicability of research overall.^17^^,^^20^^,^^33^, ^34^, ^35^

Essential information required to critically appraise a study is often missing from pathology AI conference abstracts. This presents problems in terms of gathering and synthesizing evidence from multiple studies in this field, as well as the risk of research waste and duplication. Additional challenges presented by the nature of AI studies further compound this problem. We recommend the use of the STARD for Abstracts criteria as part of the abstract submission process at conferences to improve completeness of reporting, and therefore the quality of evidence. Furthermore, consideration of areas of bias in studies of AI could be addressed in the future development or updates to guidance for diagnostic accuracy studies. We hope to see greater endorsement of these across guidelines across the international pathology community as part of our shared pursuit of the best evidence-based medicine.

## Authors contributions

All authors were involved in the conception and design of the study. CM and DT performed the data collection and analysis with advice and interpretation from PB. All authors were involved in writing the manuscript and had final approval of the submitted and published version.

## Competing Interests

P.B. led the development groups for STARD and STARD for Abstracts.

C.M. and D.T. have no interests to declare.
